# Interference between face and non-face domains of perceptual expertise: a replication and extension

**DOI:** 10.3389/fpsyg.2014.00955

**Published:** 2014-09-10

**Authors:** Kim M. Curby, Isabel Gauthier

**Affiliations:** ^1^Department of Psychology, Macquarie University, SydneyNSW, Australia; ^2^Department of Psychology, Vanderbilt University, NashvilleTN, USA

**Keywords:** perceptual expertise, interference, faces, objects, holistic processing

## Abstract

As car expertise increases, so does interference between the visual processing of faces and that of cars; this suggests performance trade-offs across domains of real-world expertise. Such interference between expert domains has been previously revealed in a relatively complex design, interleaving 2-back part-judgment task with faces and cars (Gauthier et al., 2003). However, the basis of this interference is unclear. Experiment 1A replicated the finding of interference between faces and cars, as a function of car expertise. Experiments 1B and 2 investigated the mechanisms underlying this effect by (1) providing baseline measures of performance and (2) assessing the specificity of this interference effect. Our findings support the presence of expertise-dependent interference between face and non-face domains of expertise. However, surprisingly, it is in the condition where faces are processed among cars with a disrupted configuration where expertise has a greater influence on faces. This finding highlights how expertise-related processing changes also occur for transformed objects of expertise and that such changes can also drive interference across domains of expertise.

## INTRODUCTION

Face perception is often described as a domain of perceptual expertise. Our skill with faces manifests itself across many different tasks and is often particularly impressive for familiar faces. For example, normal adults can recognize familiar faces with accuracy >90% despite not having seen some of these faces for over 35 years (Bahrick et al., 1975). Most people are as fast to categorize an image as a “face” as they are to categorize it at an individual level (“Bill Clinton’s face”; Tanaka, 2001). In contrast, observers are much slower to categorize an image of a bird at a similar subordinate level—for example, categorizing an animal as a “cardinal” is slower than categorizing it at the basic level, “bird” (Tanaka and Taylor, 1991). But even the processing of unfamiliar faces outshines our performance with other objects in several respects. For instance, observers can retain more faces in visual short-term memory than they can other objects (Curby and Gauthier, 2007; Curby et al., 2009). Face processing is also more sensitive to subtle changes in the spatial-relations between features than object processing (Haig, 1984; Hosie et al., 1988; Kemp et al., 1990; Bruce et al., 1991).

Our skill with faces is believed to result, at least in part, from our extensive experience with them (but see Kanwisher, 2000), and to be mediated by the acquisition of a holistic processing strategy (Diamond and Carey, 1977; Richler et al., 2011a). What holistic means varies to some extent, as it is sometimes described as a sensitivity to configuration, a global (as opposed to local) information sampling strategy, a situation where perceiving the whole is greater than the sum of its parts, or integrality of processing for different dimensions (see Richler et al., 2012 for a review). These different meanings motivate authors to use a number of tasks to compare face and object recognition. One commonly used meaning of holistic processing is as a failure of selective attention. For instance, Young et al. (1987) asked people to name the identity of part of a face composite, and found they were unable to do so while ignoring other parts of the composite. When the composite is inverted or the composite parts are misaligned, people can more easily selectively attend to a face part. This has been replicated in variations on this original paradigm, such as in matching tasks with unfamiliar faces (Farah et al., 1998; Richler et al., 2008; Curby et al., 2013). Like several other hallmarks of face perception [e.g., the inversion effect; Rossion et al., 2002; Curby et al., 2009; the sensitivity to spatial frequency content; McGugin and Gauthier, 2010; recruitment of the fusiform face area (FFA); Gauthier et al., 2000, 2005; McGugin et al., 2012a], this kind of holistic processing has also been obtained for non-face categories when expert observers are tested, for instance for cars in car experts (Gauthier et al., 2003; Bukach et al., 2010), chess displays in chess experts (Boggan et al., 2012), and novel objects after expertise training in the lab (Gauthier and Tarr, 2002; Wong et al., 2009).

Based on the idea that face processing can be understood as a kind of expertise, it has been suggested that it may share more resources with the processing of other objects of expertise than with typical object perception. The logic is simple: if the perceptual strategies and neural substrates were found to be similar, this may lead to interference when two categories of expertise are processed simultaneously. We originally tested this prediction in an electrophysiological study using the composite paradigm to measure holistic processing and a neural marker of expertise, the N170. We recruited participants with a range of car-recognition skills, from none to extensive, and developed a paradigm in which participants processed faces and cars concurrently (Gauthier et al., 2003). Participants matched the bottom parts of face and car composites, while faces and cars alternated. In this 2-back part-matching task, we were able to measure holistic processing of faces when presented in two different contexts: (1) Among normal cars, which car experts were found to process more holistically than car novices, and (2) among cars with inverted tops, which car experts did not process holistically. Therefore, we expected that holistic processing of normal cars would compete with that of faces, only in car experts. Indeed, we found that faces in the context of normally configured cars were processed less holistically [i.e., there was less influence from the to-be-ignored (top) part on bottom judgments] than those presented in the context of cars in a transformed configuration (tops inverted). These results suggested a functional overlap between face and car processing that is related to an individual’s level of expertise with cars.

Since our original study, there have been other studies providing evidence of interference between faces and objects of expertise using event-related potential (ERP; Rossion et al., 2004, 2007), functional magnetic resonance imaging (fMRI; McGugin et al., 2014), or other behavioral paradigms (McKeeff et al., 2010; McGugin et al., 2011). There are also other studies suggesting that the processing of faces and words may compete during development and influence their lateralization in the brain (Dehaene and Cohen, 2011; Dundas et al., 2013). However, Gauthier et al. (2003) is the only study that looked at functional overlap specifically in holistic processing. The goal of the present study was to replicate this finding (Simons, 2014) and to explore its underlying mechanisms using baseline conditions that were not used before.

The measurement of holistic processing is relatively complex, with holistic processing in the composite task quantified using a difference score between two indices of discriminability (each a *d*′ measure that depends on a hit-rate and a false-alarm rate). The design used by Gauthier et al. (2003) not only requires holistic processing to be measured for both faces and cars concurrently, but also the calculation of an interference index which is the relative amount of holistic processing for faces in two different car contexts. A significant correlation of this interference index with a measure of car expertise can be obtained for several reasons, and our goal was to try to understand what led to this correlation.

The interference index is a difference of differences: the congruency effect for faces in the context of normal cars – the congruency effect for faces in the context of transformed cars, with each congruency effect being a difference score itself. One concern with correlations with difference scores is that the variance captured in a correlation can come from the main condition, the control condition, or both (e.g., DeGutis et al., 2013). This is not necessarily a problem, depending on the construct measured, but it can lead to misleading interpretations. The difference score that yields the congruency effects is central to the definition of holistic processing as a failure of selective attention and authors generally do not consider its components further (DeGutis et al., 2013; Richler and Gauthier, 2014). In contrast, the difference between holistic processing in the two contexts is not a unitary construct. The original prediction is that interference occurs in one context (when both faces and cars are shown in their normal configurations) and that it is not found in the other context (when cars are transformed so that they do not engage expert processes in car experts).

Here we first replicated the original finding (Experiment 1A), then unpack the effect in ways that were not explored before. In particular, we ask whether interference as a function of car expertise is attributable to the condition in which faces are shown in a normal car context. To preview our results, we find that it is not, and so we set out to compare the effect to different baseline conditions, in the hope of clarifying the locus of the effect. In Experiment 1B, we test our prediction that when comparing to a baseline with no irrelevant parts, it would be the car experts’ performance that would show interference, and not car novices. The baseline will also help characterize the interference as facilitation in congruent trials or interference in incongruent trials. Finally in Experiment 2, we replace cars with novel objects to assess whether the interference between two domains can be obtained when performance on the interleaved task is matched, but does not tap into expert processes.

## EXPERIMENT 1A

To assess the robustness of this effect, we first conducted a replication of the study previously reported in Gauthier et al. (2003).

### METHOD

#### Participants

Thirty-five individuals with normal or corrected-to-normal vision volunteered to participate for payment: 17 self-reported as car experts and 18 as novices (six women, one reporting as a car expert). The rights of the subjects were protected according to a protocol approved by Vanderbilt University’s Institutional Review Board. The data from two novices were later discarded, one because of poor overall performance in the task (54%) and the other because he was an outlier (>3 SD) on our interference index (see design and procedures).

#### Stimuli

**FIGURE 1:**
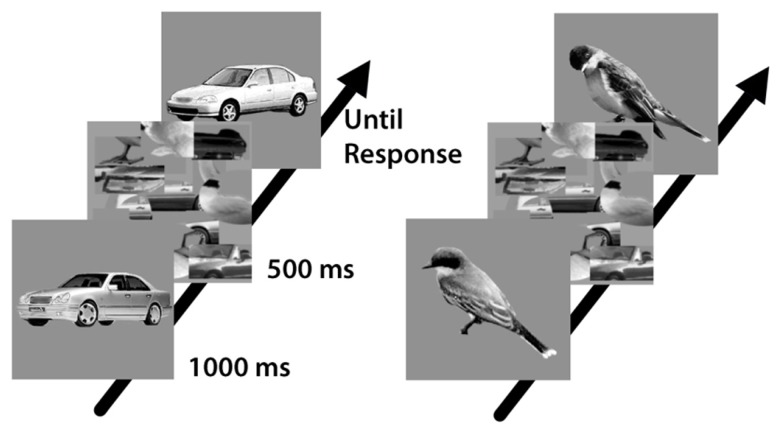
**Sequential matching paradigm with images of cars and birds was used to measure car expertise (relative to a baseline of performance in bird matching; Gauthier et al., 2000, 2005)**.

**FIGURE 2:**
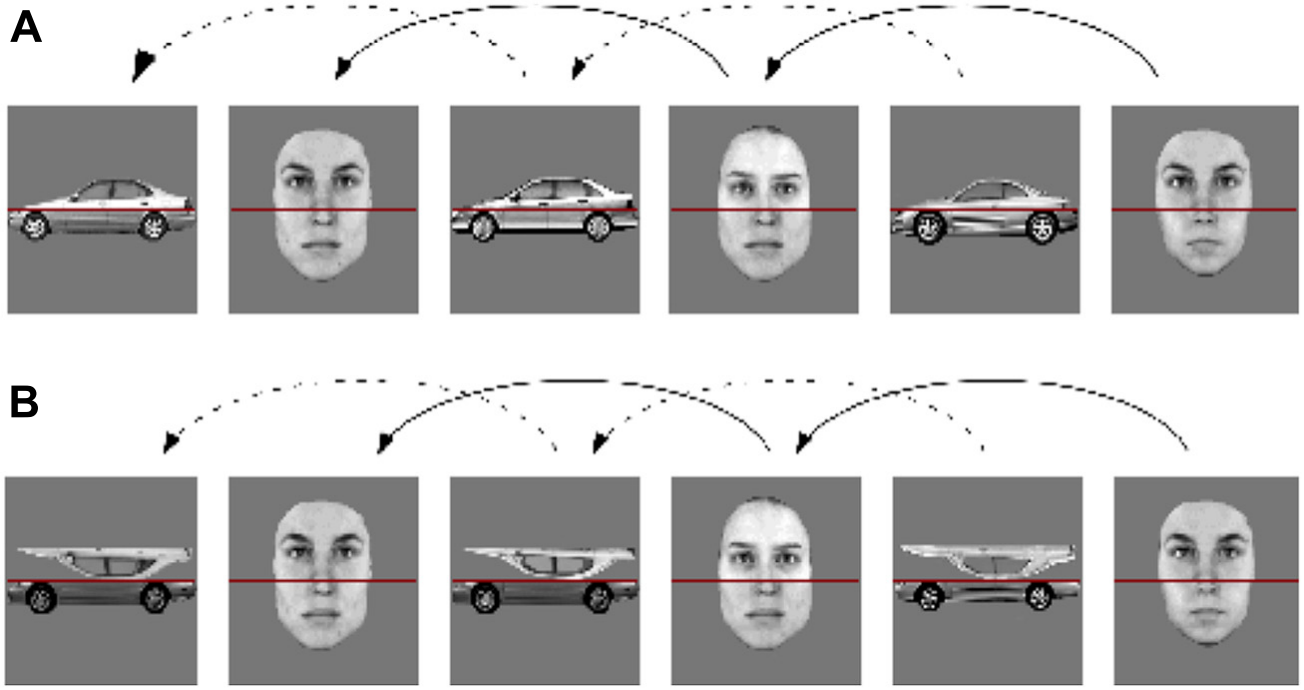
**2-back interleaved part-matching task designed to measure holistic processing for cars and for faces in a situation where the processing for both categories overlaps in time (Gauthier et al., 2003).** Composite faces were interleaved with composite cars in either **(A)** an intact (familiar) or **(B)** transformed (tops inverted) configuration.

For the car expertise test, 120 pictures of different year and/or model cars and 120 pictures of different bird species from viewpoints varying from profile to three-quarter view were used (**Figure 1**). In the interference task, 336 grayscale (256 × 256 pixels) composite images of cars (profile) and faces (front view) made out of the top and bottom of different original images (64 faces and 64 cars) were used (**Figure 2**; see Gauthier et al., 2003). All images had a horizontal red line covering the seam between the two parts. In half of the car images the top part was inverted. The stimuli were presented on a 19-inch monitor with a display resolution of 1280 × 960 pixels. Participants sat ∼70 cm from the screen. The position of participants’ heads was not fixed.

#### Design and procedure

Self-report of expertise is not always a good predictor of performance (Diamond and Carey, 1986; Rhodes and McLean, 1990; McGugin et al., 2012b) and thus participants were required to perform a car expertise test (**Figure 1**; see Gauthier et al., 2000) in addition to the main interference task (**Figure 2**). This car expertise test yielded a quantitative estimate of their perceptual skill with cars relative to their skill with a baseline category, birds. Over 224 trials, participants matched sequentially presented, 256 × 256-pixel, grayscale images of cars and birds on the basis of their model or species (see **Figure 1**). The first image was presented for 1000 ms and was followed by a mask for 500 ms. Then the second image appeared and remained until either the subject made a response or 5000 ms had passed. Performance on the bird trials provided a baseline measure for individual differences at subordinate-level matching for a category of familiar objects in the absence of expertise. As in Gauthier et al. (2003), a car expertise score was calculated by subtracting the *d*′ for birds from the *d*′ for cars for each individual.

In the interference task, participants performed 1020 trials (60 practice, 960 experimental) in which an image was presented centrally either for 1500 ms or until they made a response. Images alternated between car and face composites (see **Figure 2**). Participants pressed a key indicating whether the bottom of the current image was the same or different from the last image of the same category, triggering the presentation of the next image. Thus, participants performed a 2-back part-matching task in which they were told to always ignore the top of cars and faces.

Similar to the paradigm used in Gauthier et al. (2003), car configuration was manipulated to influence the extent to which they should elicit HP in car experts: (i) an upright normal condition (**Figure 2A**) and (ii) an inverted-top condition (**Figure 2B**). The two interference conditions alternated in 15 blocks of 60 trials (a break was given every 30 trials). Half of the trials were congruent, where the information from the to-be-ignored top parts would lead subjects to make the same judgment as the information from the attended bottom part (when compared to the 2-back stimulus from the same category). The other trials were incongruent; information from the to-be-ignored top part would lead subjects to make the opposite judgment as the attended bottom part.

Notably, if participants could follow instructions and completely ignore the top part of composites when making 2-back judgments on the bottom part, it would make no difference whether the top part was congruent or incongruent with the correct response for the bottom part. Thus, the degree to which the irrelevant top parts influence judgments about the task-relevant bottom part provides an index of HP (as in Wenger and Ingvalson, 2002, 2003; Gauthier et al., 2003).

### ANALYSIS

Face-matching trials performed in the context of cars with inverted tops and those performed in the context of cars with upright tops were split into congruent and incongruent trials. The car trials were also split into congruent and incongruent trials. Sensitivity (*d*′) was calculated for the congruent and incongruent trials for each of the face (upright car-top context, inverted car-top context) and car (upright tops, inverted tops) conditions. HP was operationalized as the sensitivity for congruent minus incongruent trials (HP = *d*′_congruent_ – *d*′_incongruent_).

The Interference index was then calculated by subtracting the amount of HP for faces in the high interference condition, where the faces were processed in the context of upright cars, from that in the low interference condition, where faces were processed in the context of cars with inverted tops. This index provides a measure of the change in HP of the faces due to manipulating the configuration of the cars. Because modifying the configuration of objects of expertise has been shown to impact HP (Young et al., 1987), this index will allow us to detect any trade-offs in HP between the two tasks. Crucially the faces and the cars presented in both conditions were identical except for the orientation of the top (irrelevant) part of the cars, and therefore any difference in HP of the faces between the two conditions can be attributed to the context within which the faces were processed.

### RESULTS

Expertise in car recognition varied from none to extensive. There was little variability in bird-matching performance (none of our participants reported any special experience with birds and bird-matching performance was low, ranging from 0.12 to 1.38 *d*′, consistent with their self-report) compared to car-matching performance where *d*′ scores ranged from 0.37 to 3.76. Consistent with past work, there was a modest, non-significant, correlation between car and bird scores (*r*_32_ = 0.28, *p* = 0.10).

Even though participants were never asked to make a judgment about the top, they apparently could not refrain from processing both faces and cars holistically (see **Table 1**). This bias was stronger for faces than cars (*t*_32_ = 7.941, *p* < 0.0001, *d* = 2.81), this is likely a result of more extensive expertise with faces (Gauthier and Tarr, 2002). Normal cars were processed more holistically than transformed cars, a manipulation check (HP expressed as Δ*d*′ for normal cars: 0.86 ± 0.07, for transformed cars: 0.42 ± 0.09, *t*_32_= 3.601, *p* < 0.002, *d* = 1.27).

**Table 1:** Sensitivity (*d*′ and % accuracy) and the derived measures of holistic processing and interference for subjects in Experiment 1A divided in a novice and expert group according to a median split on the measure of car expertise.

	Congruent	Incongruent	Holistic processing (congruent–incongruent)	
**Car sensitivity**				
*Novices*				
Inverted car tops	1.57 (77.1)	1.03 (68.8)	0.54	
Normal cars	1.39 (74.9)	0.62 (62.0)	0.77	
*Experts*				
Inverted car tops	1.90 (81.6)	1.61 (77.6)	0.29	
Normal cars	1.93 (82.6)	0.97 (68.0)	0.96	
**Face sensitivity**				Interference index (HP new context–HP old context)
*Novices*				
Transformed car context	1.89 (81.9)	0.76 (64.8)	1.12	
Familiar car context	1.80 (80.7)	0.48 (59.5)	1.32	
				-0.20
*Experts*				
Transformed car context	2.41 (87.3)	0.82 (65.9)	1.58	
Familiar car context	2.07 (83.1)	0.63 (62.4)	1.44	
				0.14

**FIGURE 3:**
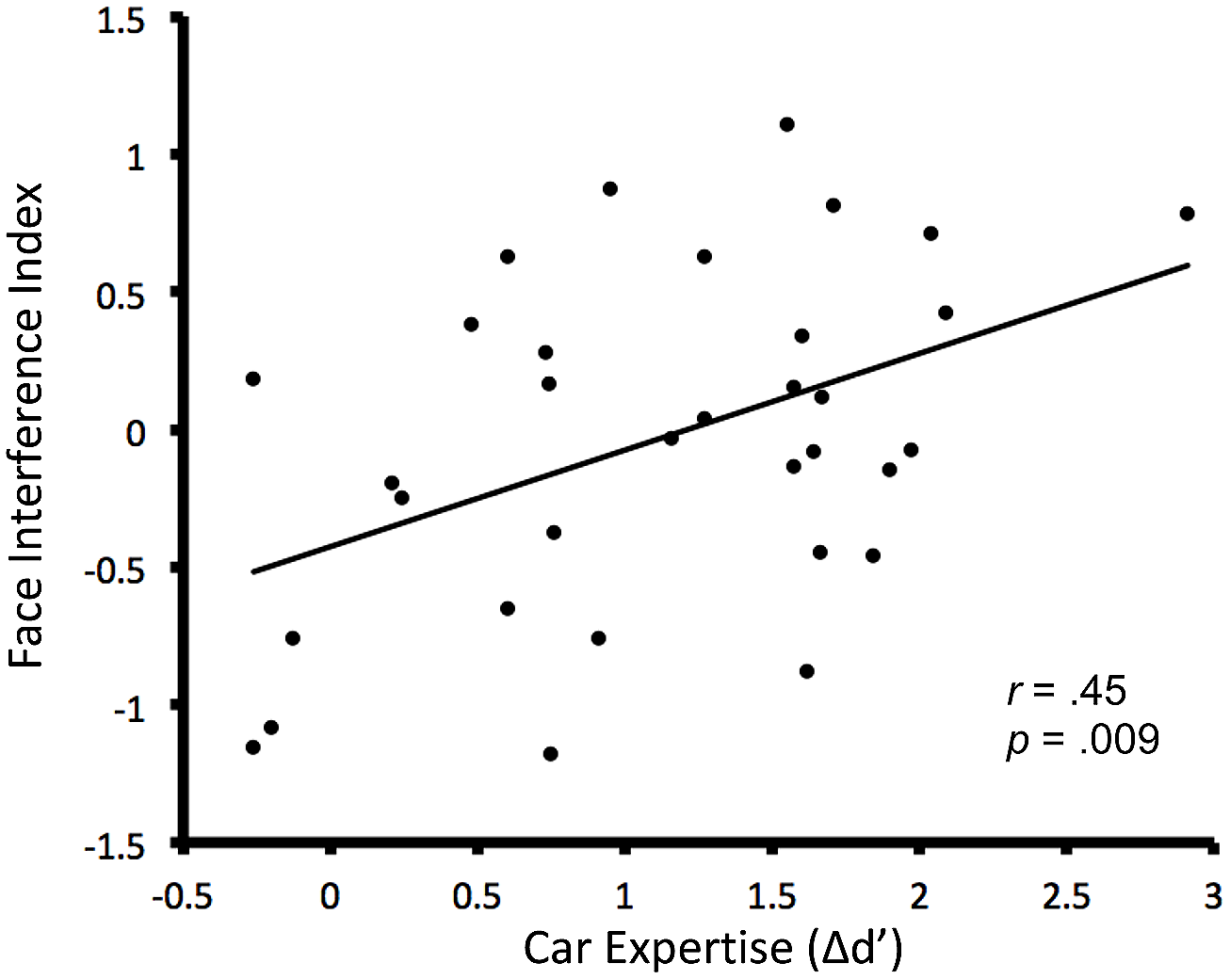
**Relationship between the participants’ car expertise score (sensitivity for cars minus their sensitivity for birds in the expertise test) [Δ*d*′] and the face interference index defined as the amount of holistic processing for faces when cars were in a new configuration compared to the holistic processing of faces when cars were in their normal configuration**.

Faces seen in the context of normal vs. transformed cars led to approximately the same degree of HP when expertise was ignored (HP for faces seen in the context of normal cars: 1.38 ± 0.08, for faces seen in the context of transformed cars: 1.35 ± 0.10, *t*_32_ = 0.289, *p* = 0.77, *d* = 0.10). Critically however, when car expertise was taken into account, HP for faces depended on the configuration of the interleaved cars; as predicted, individuals with higher levels of car expertise had higher interference indexes (HP for faces seen in the context of transformed cars minus that for faces seen in the context of normal cars; *r*_32_ = 0.45, *F* = 7.67, *p* = 0.009; **Figure 3**)^1^.

Intriguingly, while most experts had a positive interference index, suggesting that they processed faces more holistically in the context of cars in a modified, rather than intact, configuration, most novices had a negative interference index. This suggests that car novices actually processed faces more holistically in the context of cars in an intact, rather than modified, configuration. We also looked at the correlations between car expertise and each of the two face conditions separately: car expertise did not predict HP for faces viewed among normal cars (*r*_32_ = -0.01, *p* = 0.96), whereas it predicted HP for faces viewed among transformed cars (*r*_32_ = 0.45, *p* = 0.007), a significant difference (Steiger’s *Z* = -2.05, *p* = 0.04). These findings suggest that the interference between faces and cars occurs in the transformed car condition, which was not the original prediction.

### DISCUSSION

Consistent with previous findings, Experiment 1A provided evidence of interference between face and car processing as a function of expertise with cars (Gauthier et al., 2003). These data demonstrate that interference across different domains of perceptual expertise, as measured via the impact on an established index of holistic perception, is a robust and replicable effect. However, the effect was unexpectedly driven by an interaction between faces and transformed cars. That is, car novices and car experts differed more in their face processing in a transformed car context than in the familiar car context (see **Table 1**). This is inconsistent with the original predictions that motivated this task (i.e., that in car experts, face recognition would compete most with the processing of whole cars that produce more HP).

One reason why these findings are relatively difficult to interpret is that car experts may differ from novices in the amount of HP in several ways. For instance, car experts may show more HP of cars than car novices because they experience more interference from incongruent to-be-ignored parts, more facilitation from to-be-ignored parts, or both. Likewise, the difference in HP of faces by car experts and car novices in the different car contexts can also be driven by a difference in facilitation, interference or both. In addition, while we measured the effect of car expertise as a continuous variable, it may seem reasonable to predict that relative to a baseline condition where holistic processing is not implicated, it would be performance for the car experts, and not for the novices, that would show an interaction with face processing. But because our initial prediction that faces and normal cars would be mainly responsible for this interaction was not supported, we decided to better characterize the effect. While the theoretical significance of whether facilitation and/or interference are critical in this interaction across domains is unclear at the moment, the proposed link between expertise and holistic processing makes a strong prediction that novices should not be affected by the presence of the irrelevant part, while car experts should.

Therefore, in Experiment 1B, we tested new car experts and novices to provide baseline conditions without to-be-ignored parts. These baselines will be used to estimate whether car experts and car novices in Experiment 1A differ most in facilitation from congruent to-be-ignored parts, or interference from to-be-ignored parts.

## EXPERIMENT 1B

To ask whether the expertise effects observed in Experiment 1A influence facilitation, interference, or both, we presented participants with only the bottom half of each image. This experiment provided baseline measures to which the sensitivity of congruent and incongruent trials could be compared.

### METHOD

#### Participants

Twenty-two (six females) subjects with normal or corrected-to-normal vision and varying levels of car expertise who had not performed in Experiment 1A participated in this study for payment or course credit. The rights of the subjects were protected according to a protocol approved by Vanderbilt University’s Institutional Review Board.

#### Materials

Stimuli were the same as Experiment 1A except the top half of each was removed.

#### Design and procedure

The procedure was the same as in Experiment 1A except only the bottom half of the car and face images were presented (i.e., the parts above the red line in **Figure 2** were omitted).

### ANALYSIS

The data were divided into expert and novice groups based on participants’ car expertise indices (i.e., the difference between car- and bird-sensitivity in the car expertise test; see Experiment 1A for details). Consistent with previous studies, we defined experts as individuals with a *d′* for cars >2 and a car expertise index (car *d′*–bird *d′*) >1 (Gauthier et al., 2000). The data from Experiment 1B was also divided into groups of experts and novices. To facilitate comparison across Experiments 1A and 1B, the average expertise index for each of the groups was matched across the two studies. This was done in such a way as to exclude data from as few participants as possible, based only on their car expertise scores. The resulting mean Δ*d′* and sample sizes (Experiment 1A/Experiment 1B) for the groups were as follows: Expert 1.83 (*N* = 14)/1.81 (*N* = 9) and Novice 0.37 (*N* = 14)/0.36 (*N* = 9).

In general it is more statistically powerful to use car expertise as a continuous variable than as a dichotomous variable. However, because Experiments 1A and 1B include different subjects who are not matched individually but in groups of novices and experts, we report next a series of ANOVAS on Experiment 1A alone and relative to the baselines obtained in Experiment 1B in which car expertise is treated as a dichotomous variable.

### RESULTS

#### ANOVA comparing expert and novice performance in the 2-back (tops present) task (Experiment 1A)

A 2 (group; novice, expert) × 2 (car top context; upright, inverted) was performed on the HP measures for the new groups created from the data reported in Experiment 1A. Consistent with a role of car expertise in modulating the effect of car context on face processing, as revealed in the correlation analysis reported above, a significant interaction emerged between group and car context, *F*(1,26) = 5.97, *p* = 0.022, η^2^ = 0.064. Car context had a different effect on face processing depending on car expertise; for novices, inverting the top of the cars led to a decrease in HP for faces. In contrast, for car experts, inverting the top of the cars led to an increase in HP for faces. There was also a marginally significant effect of group, *F*(1,26) = 4.13, *p* = 0.053, η^2^ = 0.090, suggesting that car experts in general processed the faces more holistically. There was no main effect of car context on HP of faces (*F* < 1). Planned *t*-tests showed that while HP for faces among normal cars failed to differentiate between car experts and novices, *t*(26) = 0.30, *p* = 0.77, *d* = 0.12, car experts processed faces shown among transformed cars more holistically than car novices, *t*(26) = 2.76, *p* = 0.01, *d* = 0.48.

**FIGURE 4:**
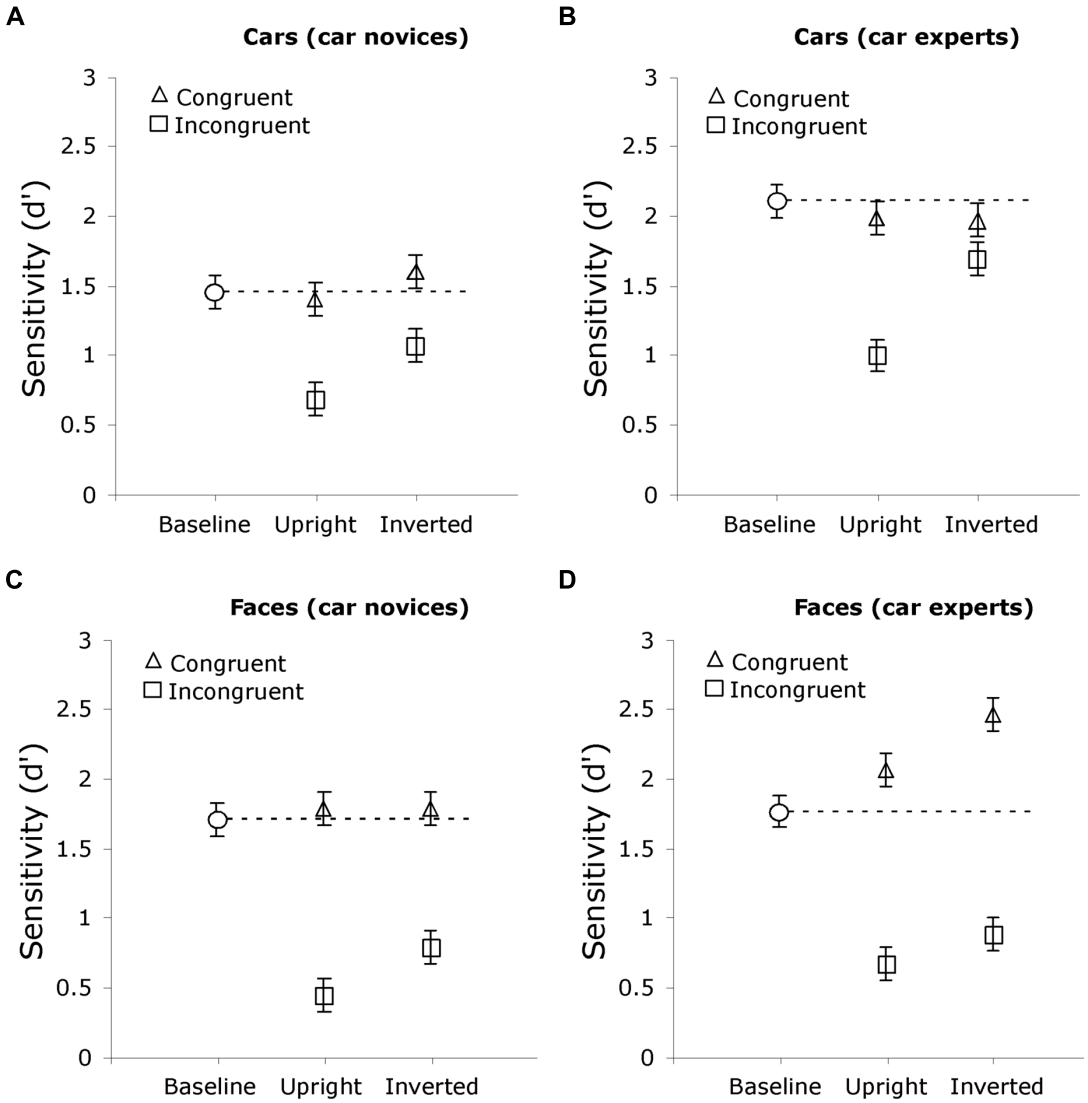
**Sensitivity for car, **(A,B)**, and face, **(C,D)**, matching judgments of bottom halves of composites for groups of car novices, **(A,C)**, and experts, **(B,D)**, in Experiment 1A.** In each graph, the results from the two conditions in Experiment 1A (when the top of cars were upright and when they were inverted) are plotted with the baseline provided by matched groups of novices and experts in Experiment 1B, matching the bottom halves of faces or cars with no top half present. Error bars show the standard error of the mean.

Planned comparisons on HP for cars revealed more HP for upright cars than cars with inverted tops in car experts, *t*(26) = 3.58, *p* = 0.001, *d* = 1.40, but not in novices, *t*(26) = 1.03, *p* = 0.31, *d* = 0.40 (see **Figures 4A,B**). In fact, HP was significantly different from 0 in all car conditions (all *p*s ≤ 0.0005) except for cars with inverted tops in car experts (*p* = 0.13). This suggests that inverting car tops made them easier to ignore for car experts.

#### ANOVA comparing expert and novice performance in 2-back half (tops absent) task (Experiment 1B)

A 2 (group; experts, novices) × 2 (category; faces, cars) ANOVA on the results for the baseline task (no-top) revealed a significant interaction between group and category, *F*(1,16) = 7.34, *p* = 016, η^2^ = 0.041. As expected, car novices and experts did not differ in their performance matching face bottoms (*p* > 0.05), but car experts were better at matching car bottoms than novices (*p* < 0.001).

#### Comparison of Experiment 1A against baselines from Experiments 1B

**Figure 4** illustrates the results in Experiment 1A in each condition, for car novices and car experts, relative to the baselines obtain in Experiments 1B where no to-be-ignored parts were present. As we have seen, these baselines indicate when participants performed the task with only the to-be-attended parts, car novices and car experts only differed in their processing of car parts, being better in car experts. Now we use these baselines to interpret the results of Experiment 1A and specifically ask in what way car novices and car experts differ.

The results of planned *t*-tests comparing sensitivity (*d*′) in the top-present congruent and incongruent conditions (from Experiment 1A) with their respective no-top baselines (from Experiment 1B) reveals that HP during the top-present task was mainly due to interference from incongruent top halves rather than facilitation from congruent ones (**Figure 4**). Incongruent conditions led to lower sensitivity than their respective baselines (all *p*s < 0.05) in all cases except for cars with inverted tops in both groups (*p*s < 0.05). In contrast, the only condition with significant facilitation was for faces seen in the context of cars with inverted tops, in car experts (*p* < 0.05).

#### Summary and discussion

Car experts experienced less interference than car novices for transformed cars (**Figures 4A** vs. **B**), while they experiences more facilitation from congruent face tops processed in this context (**Figures 4C** vs. **D**).

One account of these results is that because car experts processed cars in a transformed configuration less holistically than regular cars, they therefore recruited less HP resources. Therefore, once the top part of the car is inverted it can be effectively ignored by car experts.

Because the car context was manipulated, while the face task was the same in both car contexts, and because car novices and car experts did not differ in their processing of face parts in a car context when there was no to–be-ignored part, it is reasonable to infer that this is what led to facilitation by congruent face parts in the transformed car context. Exactly why this happened though is unclear but it is plausible that the transformed car context allowed car experts to reduce executive control when car tops were easier to ignore, and as a result also exerted less effort trying to ignore face tops.

Most experiments using the composite task have not used a baseline condition to examine facilitation/interference, but when it has been used, the congruency effect obtained for aligned faces was generally due to interference from incongruent parts, without significant facilitation (Richler et al., 2008). This is consistent with what we observed here in car novices (even if the baseline was obtained in a different group). This highlights how abnormal the processing of faces among transformed cars was in our car expert participants.

It should be noted that the choice of a baseline condition such as the isolated relevant parts used in Experiment 1B can be difficult. In the comparison between Experiments 1A and 1B, there are differences in stimuli (parts vs. composites) and in task requirements (requirement to selectively attend or not).

Given the complexity of the 2-back interleaved dual task, it is possible that other task components unrelated to HP and affected by car expertise, such as more general effects related to the executive control and/or short-term memory load demands of the task, play a role in producing these effects. Experiment 2 investigates an alternative account of the interference between different expert domains, assessing whether explanations appealing to contributions from these more general effects can be ruled out.

## EXPERIMENT 2

Our proposed account of why car experts showed more facilitation from to-be-ignored congruent face parts in the context of transformed cars points to how car experts were better able to ignore inverted car tops. Experts showed no congruency effects of inverted car tops while novices did. Non-face objects are not processed holistically in the composite paradigm, which generally means that they do not show more of a congruency effect in a normal than transformed (typically misaligned) configuration. However, there is sometimes a small but significant congruency effect for non-face objects that is not modulated by configuration (e.g., Wong et al., 2009; Richler et al., 2011b). There are situations where training has a main effect of reducing this congruency effect for stimuli in a transformed configuration (Chua et al., 2014), similar to the difference between our car novices and experts in Experiment 1A.

The absence of holistic processing for cars with inverted tops among car experts, but not novices, suggests an alternative account of the interference between face and car processing that is unrelated to expertise. Because it was the transformed car context that drove the interference effect, it is possible that the same interference effect would be observed when faces are processed in the context of any object category that does not show a congruency effect, regardless of expertise. This would suggest that an alternative account, grounded in the more general demands of the two concurrently performed tasks, rather than in participants’ expertise, would better explain this interference.

In the original paradigm, the condition where car novices process faces in the context of normal cars should had offered a test of this hypothesis. However, there was a significant congruency effect for cars in car novices here, perhaps in part because they had some non-negligible experience with cars (this congruency effect could also be amplified by cars being processed in the context of faces, see Richler et al., 2009).

**FIGURE 5:**
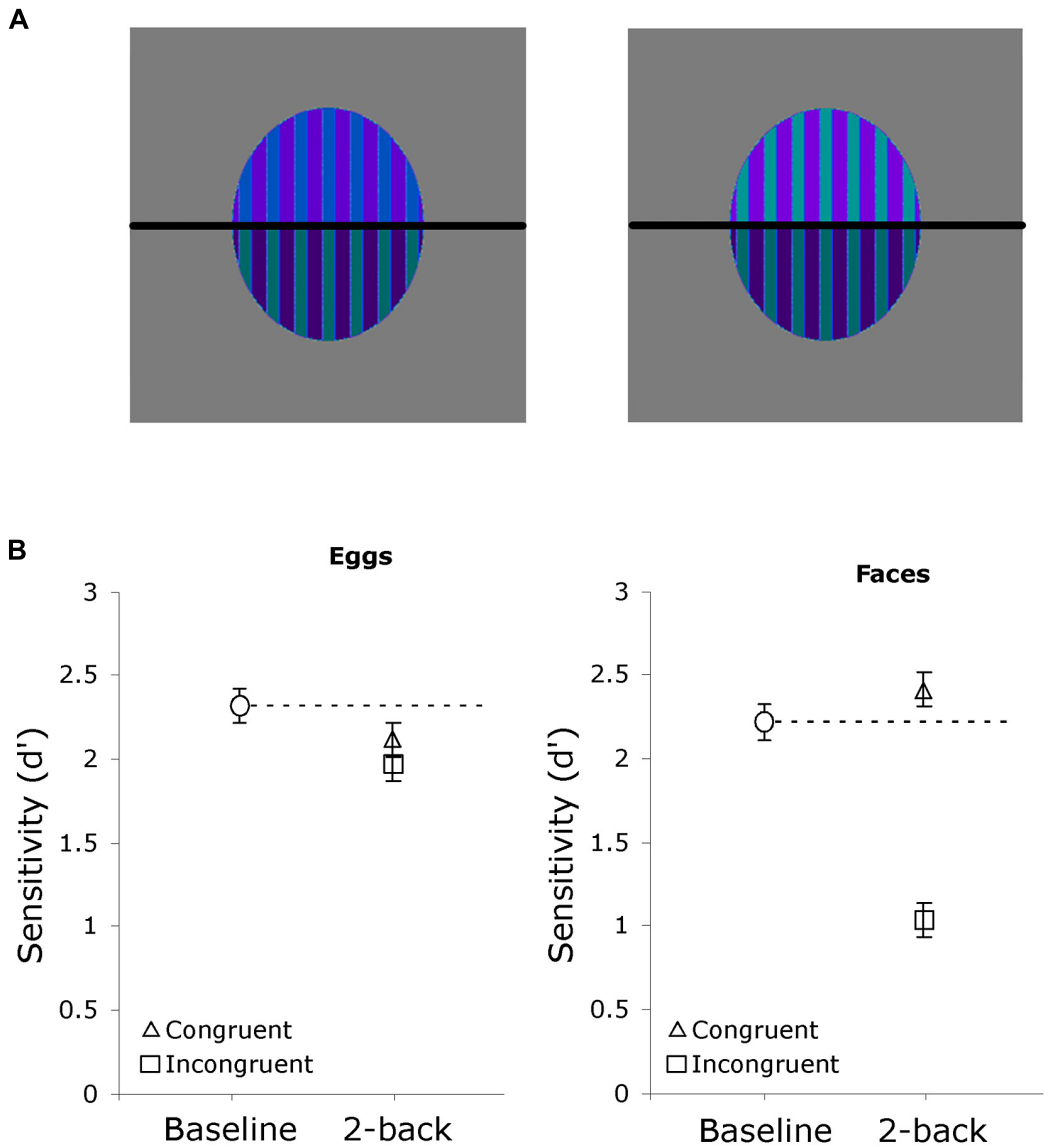
**Results for Experiment 2. (A)** Examples of egg stimuli, this pair has the same bottom but incongruent tops. **(B)** Sensitivity for egg and face judgments in Experiment 2. The results of congruent and incongruent trials are plotted against the baseline obtained from the same subjects performing the same task with no top half present.

Therefore, to test this hypothesis, we designed simple and unfamiliar stimuli in an oval shape with parts defined by colored gratings (varying in both hue and luminance, see **Figure 5A**) and in a pilot experiment, we verified that their processing in the composite task did not produce any congruency effect. Here we ask whether faces processed in our dual task with these “egg” stimuli would produce the same facilitation as observed in Experiment 1A. If the increase in the facilitation component of HP for faces processed in the context of transformed cars is simply due to the fact that the to-be-ignored parts of these transformed objects are easy to ignore, then we should observe it here. In contrast, if facilitation is not observed, this would indicate that the interaction between selective attention to faces and the car context is specifically dependent on car expertise.

### METHOD

#### Participants

We recruited fourteen volunteer participants (seven females), to match the size of the car expert group in Experiment 1. The rights of the subjects were protected according to a protocol approved by Vanderbilt University’s Institutional Review Board.

#### Stimuli

Face stimuli were the same as in Experiment 1. Stimuli were constructed from 64 oval shapes or “eggs” approximately the same size as the face stimuli with 15 vertical stripes of two alternating colors. Twelve different colors were used, selected from the Adobe Photoshop© palette to be similar but still distinguishable (all shades of blue, green or purple). The 64 original eggs were split in half and recombined (in the same manner as the face stimuli) to create 128 composite eggs used in the experiment (see **Figure 5A**).

#### Design and procedure

The procedure was identical to that of Experiment 1, except that whole faces were processed in only one context, that is the orientation of the top part of the egg was not manipulated (**Figure 5A**). The same participants also performed a baseline task with no tops on faces or eggs (as in Experiment 1B). There were an equal number of top-present and no-top trials, presented in four blocks of 240 trials. The two conditions alternated and their order was counterbalanced across participants. As in Experiment 1, participants performed 2-back judgments on the bottom half of all images.

### RESULTS AND DISCUSSION

As expected, the eggs produced a pattern of sensitivity very similar to that of cars with an inverted top in Experiment 1 (**Figure 5B**). There was no significant HP for eggs, *t*(13) = 1.21, *p* = 0.25, *d* = 0.67. Sensitivity for the egg baseline did not differ from the baseline for cars among car experts in Experiment 1, *t*(21) = 0.77, *p* = 0.45, *d* = 0.34. Sensitivity for eggs was also not significantly different from that for cars with inverted tops among experts in Experiment 1, both in the congruent, *t*(26) = 0.68, *p* = 0.50, *d* = 0.27, and incongruent conditions, *t*(26) = 1.16, *p* = 0.26, *d* = 0.45.

However, unlike in Experiment 1, this context did not lead to facilitation for congruent face trials. There was no significant facilitation (congruent trials relative to no-top baseline) for faces processed in the context of eggs, *t*(13) = 1.97, *p* = 0.07, *d* = 1.09. Comparing with Experiment 1, the estimate of facilitation from faces among eggs was both indistinguishable from that obtained from car novices matching faces among transformed cars, *t*(26) = 1.27, *p* = 0.22, *d* = 0.50, and significantly less than that obtained from car experts matching faces among cars with inverted tops, *t*(26) = 2.39, *p* = 0.02, *d* = 0.94.

The results of Experiment 2 suggest that it is not the lack of HP from the transformed car context *per se* that led to facilitation from congruent face trials in Experiment 1 as the egg stimuli were also not processed holistically, yet did not impact holistic face perception. Further, the findings of Experiment 1 also demonstrate that the interferences was not simply a general effect of participants car expertise (because in the upright car condition there was no facilitation from the to-be-ignored part for faces) nor because faces were processed among cars (because facilitation for faces was not obtained in car novices). Rather, face HP was specifically influenced by the concurrent processing of transformed objects of expertise.

## GENERAL DISCUSSION

Our results replicated the interference between holistic processing of faces and cars observed in Gauthier et al. (2003). Having found that the interaction depended on the processing of faces among transformed cars, we investigated these effects further by using a dual-task with isolated parts to partition congruency effects into interference from incongruent parts and facilitation from congruent parts. We found that car expertise was associated with less interference from incongruent car parts, and that in this context, congruent face parts produced more facilitation. Finally, we showed that this facilitation effect for faces was not obtained in another dual task with objects that produced no congruency effect, suggesting that the interaction depends on expertise.

Our results highlight the fact that transformed objects of expertise can lead to effects that are distinct from control objects. While there has been much more focus on how the processing of whole objects of expertise is special (both faces and objects), there is no question that experts also process parts differently. This is most clearly shown in our results by the advantage of car experts on car novices for car part performance. There are other examples of transformed objects of expertise producing effects that are distinct from control objects. For example, the ERP response (N170) to inverted faces is larger than the response for upright faces and delayed by 10 ms, while other objects elicit a response of much smaller amplitude and invariant to orientation (Rossion et al., 2000). This is also found in subjects who have been trained with non-face objects (Rossion et al., 2002). It is possible that these responses triggered by transformed objects of expertise index a mechanism that can interfere with expert processing of objects from another category.

Recent findings from studies of other domains of expertise, such as chess, that have also been shown to result in increased HP as indexed via the composite task, support the suggestion that transformed stimuli of expertise can trigger stronger responses in brain regions linked with perceptual expertise than their intact versions. For example, greater FFA activation was found in response to chess stimuli among chess experts when the structure of the chess stimuli was distorted compared to intact (Bilalić et al., 2011). Further, recent findings suggest that disrupted objects of expertise, such as the transformed cars used here, may trigger a search for structure or meaningful chunks by experts that appears to also involve a frontal-parietal network (Bartlett et al., 2013; Rennig et al., 2013; see also Bor and Owen, 2007). These existing findings, and the frequency with which transformed objects of experts (inverted, scrambled, misaligned etc.) are used as a control or comparison stimulus category, highlight the importance of further studies exploring the processing of such objects among experts.

Another possibility is that the expertise-related interference between face and car processing primarily reflects an attention-based effect. For instance, because car experts can more easily selectively attend to the bottom part and thus ignore the (task-irrelevant) top part in the transformed car condition, they may “relax” control of their attention in this task-context, resulting in more intrusions of congruent face parts. The effect could be carried by facilitation because interference from incongruent face parts may already be strong to start with. Our egg control task suggests that such facilitation is not observed in any situation where the to-be-ignored part is easily ignored – perhaps a certain degree of fluency with the relevant parts is also required. This account is obviously quite speculative and will require further testing.

Although this study cannot reveal where in the brain interactions between faces and cars may occur, our task is likely to engage parietal and frontal areas implicated in short-term memory (Goldman-Rakic, 1987; Belger et al., 1998) as well as the FFA (Courtney et al., 1997; Grady et al., 1998; Haxby et al., 2000; Druzgal and D’Esposito, 2001), which is the part of the brain most associated with the idea of a “face module” (Kanwisher et al., 1997; McCarthy et al., 1997). Activity in the FFA increases directly with the short-term memory load for faces (Druzgal and D’Esposito, 2001). This region is a plausible candidate for a locus of interference obtained in dual experts for a number of reasons. In particular, it is recruited for both cars and faces in car experts (Gauthier et al., 2000, 2005; McGugin et al., 2012a); the activity in this area in response to cars correlates with behavioral measures of car expertise (Gauthier et al., 2000) and although it is not the only area to show an effect of expertise for cars, when the task demands are made more difficult, as was the case here, effects in these other regions drop whereas the expertise effect in the FFA remains (McGugin et al., in press). Finally, a recent fMRI study found that car expertise effects in the FFA survived manipulations of clutter and of divided attention, but that they were abolished when cars were presented in the context of faces, especially when the faces were also task-relevant (McGugin et al., 2014). Much work remains to be done to relate this example of competition between faces and cars in the FFA with whole objects and the present finding of interaction between faces and transformed objects of expertise during a dual-task that requires selective attention.

Other findings of expertise-dependent interference between the concurrent processing of face and non-face objects of expertise suggest that not only can faces and objects of expertise be processed in a similar manner, as well as neurally close in space and time, but the neural networks responsible for their HP may not be functionally independent (Gauthier et al., 2003; Rossion et al., 2004, 2007; McKeeff et al., 2010; McGugin et al., 2011). Importantly, it is not necessary to postulate that processing of faces and cars depend on overlapping sets of neurons to account for competition between the two domains. It is sufficient to assume that face and object processing are closer together in experts than novices in “cerebral functional space” (Kinsbourne and Hicks, 1978); cerebral functional space refers to the physical size of and distance between brain areas responsible for different functions. This only assumes that competition is more likely between neural ensembles that are more densely interconnected, and/or that are separated by fewer synapses.

In conclusion, our results are consistent with previous findings of observable interference across different domains of real-world expertise where the particular domains are proposed to rely on a common resource. This functional overlap between face and non-face domains of expertise has implications for the potential of extensive learning, as in the case of real-world expertise, to lead to a dynamic reorganization of cognitive resources. Because most normal adults possess a certain degree of expertise with faces, it may be important to consider training and application environments for real-world experts and assess the extent to which competition between different domains can impact learning and performance.

## Conflict of Interest Statement

The authors declare that the research was conducted in the absence of any commercial or financial relationships that could be construed as a potential conflict of interest.

## Footnotes

1 Here we subtracted bird scores from car scores to index car expertise, consistent with prior work with this paradigm. However, in more recent work with car experts, we have regressed out performance in a non-car task from a car task to assess domain-specific effects (McGugin et al., 2014). The partial correlation between the interference index and car *d′*, partialing out bird *d′*, was *r*_31_=0.38, *p* = 0.01).
